# How to promote orderly access to medical care: an empirical analysis based on the Chinese experience

**DOI:** 10.3389/fpubh.2026.1766341

**Published:** 2026-04-02

**Authors:** Fei Li, Pengjuan Zhang, Yueran Yang

**Affiliations:** School of Management, Shanxi Medical University, Taiyuan, China

**Keywords:** China's hierarchical diagnosis and treatment system, healthcare-seeking order analysis, health-seeking behavior, service utilization, structural variation degree analysis

## Abstract

**Objective:**

This study focuses on the implementation of China's hierarchical diagnosis and treatment system, conducting an in-depth analysis from three dimensions—healthcare service framework, policy experimentation, and service utilization patterns—aiming to provide valuable insights for the construction and improvement of an orderly medical order in other countries of the world.

**Methods:**

Utilizing data from the *China Health Statistics Yearbook* and *China National Health Service Survey Reports*, this study proposes a three-dimensional framework for China's hierarchical diagnosis and treatment system, categorizes medical institutions into three distinct levels, and systematically analyzes structural variations in outpatient and inpatient service provision across different medical institutional tiers from 2007 to 2022.

**Results:**

The analysis reveals notable shifts in healthcare-seeking patterns in China: regarding first-contact care, the proportion of urban/rural residents initiating care at primary healthcare institutions demonstrates overall contraction, while secondary hospitals show increased first-contact shares, and tertiary hospitals maintaining stable utilization around 8.0%. In outpatient services, primary institutions retain the highest annual visit volumes, yet tertiary hospitals exhibit the strongest average annual growth rate. Structural variation analysis shows both tiers are primary contributors to system-wide changes—primary institutions demonstrate pre-dominantly negative VSV (Value of Structural Variation) trends, contrasted by tertiary hospitals' consistently positive VSV. For inpatient care, tertiary hospitals display the most rapid service expansion, with sustained positive VSV since 2010 and structural contribution rates exceeding 35%. This trajectory has positioned tertiary institutions as the primary inpatient service providers.

**Conclusion:**

(1) China has established clear functional roles for different medical institutions across distinct tiers of care, thereby laying a solid foundation for orderly patient referrals; (2) The evolving distribution of healthcare services across different tiers of medical institutions in China suggests that gaps remain between the current patient referral patterns and the intended objectives of the hierarchical medical system, particularly for inpatient services; (3) China's policies to promote the hierarchical diagnosis and treatment system exhibit distinct regional characteristics, highlighting the need to further expand the implementation scope of these policies to promote the formation of an orderly healthcare-seeking pattern; (4) The role of tertiary hospitals in China's hierarchical diagnosis and treatment system requires further optimization; (5) Developing integrated healthcare delivery systems with vertically coordinated services and horizontally regulated competition among medical institutions at all levels should be a critical future direction for China's health system reform.

## 1 Introduction

In recent years, in order to optimize the problem of “difficult and expensive” medical services, “hierarchical diagnosis and treatment system” has become a new keyword in the process of China's deepening medical and healthcare reform, and has been put on the policy agenda. From 2013, the “*Decision of the CPC Central Committee on Several Major Issues on Comprehensively Deepening Reform*” proposed to improve the rational hierarchical diagnosis and treatment model, and to establish a contractual service relationship between community doctors and residents, and to 2014, Premier Li Keqiang emphasized in the government work report, “to perfect the hierarchical diagnosis and treatment system, and strength the cultivation of general practitioners, and promote the multi-disciplinary training of physicians, so that the public can enjoy high-quality medical services close to their homes “, and then in 2015, the “*Guiding Opinions of General Office of the State Council of the People's Republic of China on Promoting the Construction of the Hierarchical Diagnosis and Treatment System”* explicitly called for “establishing hierarchical diagnosis and treatment system which conforms to the conditions of the country”, “hierarchical diagnosis and treatment system” serving as an important policy tool to build an orderly medical order for residents, has emerged as a central priority in the reform and development of China's healthcare service system.

From the perspective of international studies, China's hierarchical diagnosis and treatment system aligns with the three-tier healthcare service model and gatekeeper system promoted globally. The essence of them is a medical service pattern based on cooperation in medical services among different levels and categories of medical institutions, as well as maximization of the efficiency of medical resource allocation and utilization, and refinement of patient management services (1). For example, the United States has developed a primary care-oriented health care delivery system, where primary care physicians are responsible for making initial decisions as well as making appointments with specialists for referrals to specialized facilities, which means specialists typically serve as the point of contact only for uncommon conditions, with their expertise focused on specific diseases rather than comprehensive care (2, 3). In the UK, “the gatekeeper system” constitutes a vital component of its integrated care framework. “Gatekeepers” refer to authorized primary care physicians or general practitioners responsible for coordinating access to specialist care, hospital treatment, and diagnostic testing. This system establishes a refined division between primary and secondary care, ensuring prompt access to specialized treatment for those in need (4). In France, the 2005 Health Financing Reform Act introduced a “voluntary gatekeeper” program, aimed at regulating outpatient specialist care. This policy provides financial incentives to encourage patients to first consult general practitioners rather than seeking specialists directly (4).

It can be argued that the gatekeeper system, widely adopted by the international community, emphasizes the role of general practitioners in referring patients for the ongoing management of specific diseases and conducting regular consultations for routine monitoring (2). The system facilitates high-standard healthcare development by curbing excessive medical interventions, enabling effective task division between general practitioners and specialists, allowing specialists to freely advance their professional expertise, and controlling healthcare costs (5). The implementation of China's hierarchical diagnosis and treatment system also emphasizes patients' access to medical care in accordance with the medical hierarchy system, whereby patients need to undergo initial diagnosis and treatment by primary care physicians, and then be referred to the most appropriate medical institution for proper diagnosis and treatment according to the type of disease and condition. This aligns with the international concept of orderly healthcare-seeking rooted in the “gatekeeper” system. Based on this, this study focuses on the operational status of China's hierarchical diagnosis and treatment system, and conducts a multidimensional analysis of the service system, policy practice, and patients' service utilization behaviors. The results of the study are intended to contribute to the construction and improvement of an orderly medical order in other countries of the world.

## 2 Methods

### 2.1 Model construction

**Figure 1:**
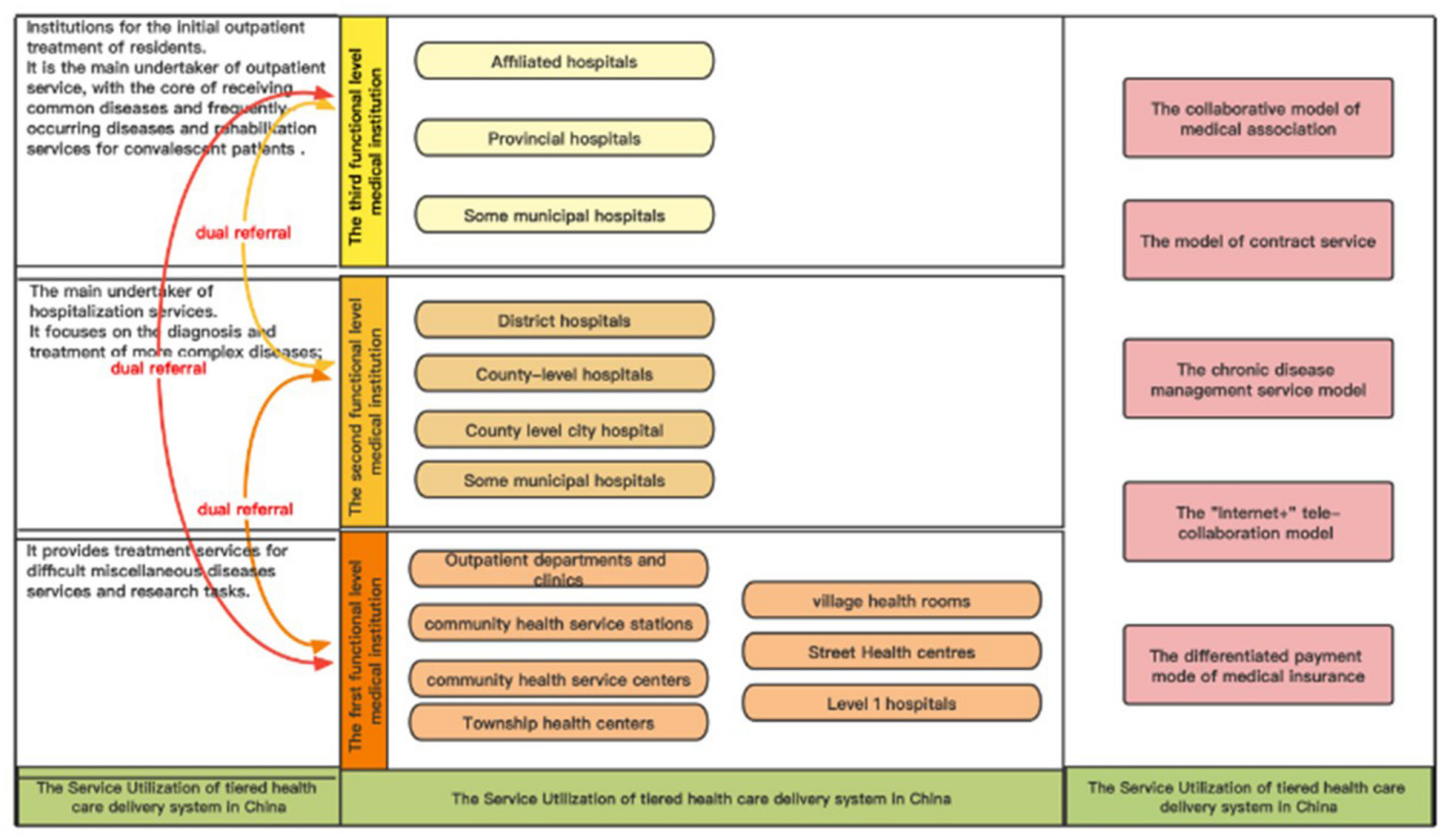
The three-dimensional structural model of hierarchical diagnosis and treatment system in China.

This study constructs a three-dimensional practice model of China's hierarchical diagnosis and treatment system, which includes the service providers, the relevant policies, and the patients' behaviors of service utilization, as shown in Figure 1. At the dimension of service providers, which forms the foundation for implementing hierarchical diagnosis and treatment system, emphasis is placed on the division of labor and collaboration among different levels of medical institutions. At each level, service providers differ in the order and functions of medical service delivery. At the dimension of relevant policies, which drives and safeguards for implementing hierarchical diagnosis and treatment system, emphasis is placed on the construction of a series of administrative policies, including resource allocation, talent training, service incentives and so on, to form a strong impetus to the division of labor and collaboration among different levels of medical service providers. At the dimension of patients' behaviors of service utilization, which represents the ultimate objective for implementing hierarchical diagnosis and treatment system, emphasis is placed on the orderly behaviors of patients' utilization of medical services, which could enhance resource utilization efficiency. In these three dimensions, service provision forms the foundation for the implementation of the hierarchical diagnosis and treatment system; policy practice plays a driving and safeguarding role in service provision; and service utilization is the ultimate focus of the hierarchical diagnosis and treatment system. All three dimensions center on the core of hierarchical diagnosis and treatment, with distinct focuses and mutual complementarity, and collectively constitute a systematic analytical framework for understanding the practice of hierarchical diagnosis and treatment.

#### 2.1.1 Dimension I: service providers

In China, the medical service providers include primary health care (PHC) institutions and hospitals (6–9). PHC institutions, which mainly provide diagnosis and treatment services for common and frequent diseases, and also engaging in health education, disease prevention, chronic disease management, and rehabilitation services, include community health service centers (stations), village clinics, township health centers, and other private clinics. Hospitals are categorized into first-level hospitals, second-level hospitals and third-level hospitals. First-level hospitals (with 20 to 99 inpatient beds) serve as grassroots facilities directly providing preventive, medical, healthcare, and rehabilitation services to communities with populations ≤ 100,000. Following the implementation of the 1997 healthcare system reform, a notable proportion of these hospitals have been restructured into community health service institutions. Second-level hospitals (with 100 to 499 inpatient beds) are regional medical institutions that provide medical care to multiple communities (with populations ≥100,000 people). These hospitals, functioning as general or specialized regional medical facilities, provide inpatient services for most diseases, and additionally undertake certain teaching and research responsibilities. Third-level hospitals (with 500 or more inpatient beds) deliver high-standard specialized medical services across regions while fulfilling higher education and scientific research responsibilities.

Taking into account the situation of medical service institutions in China, this study categorizes existing medical service providers into three levels within the hierarchical diagnosis and treatment service system. Level 1: primary healthcare institutions including first-level hospitals, community health service institutions, township health centers, village clinics, and other private clinics, primarily deliver diagnosis and treatment services for common and frequent diseases, along with rehabilitation services for patients in recovery period. Level 2: secondary-level hospitals at the county/district level, serving several communities, provide diagnosis and treatment of more complex diseases and inpatient services. Level 3: tertiary-level hospitals at municipal or higher administrative levels, serving several regions, undertake services for difficult and complicated diseases and medical research tasks.

#### 2.1.2 Dimension II: policy practice

In 2015, China introduced *the Guiding Opinions on Promoting the Construction of a Hierarchical Diagnosis and Treatment System*, which put forward the basic model of “primary diagnosis and treatment at the grassroots level, upward and downward referrals, separation of emergencies and chronic diseases, and upward and downward linkages”. Under this policy guidance, regions nationwide have actively explored mechanisms to build efficient collaborative frameworks with clear objectives and responsibilities among medical institutions at all levels, such as the medical consortium collaboration model represented by Beijing and Fujian Province, the contracted service model exemplified by Shanghai and Hangzhou, the chronic disease management service model pioneered in Zhejiang Province and Xiamen, the “Internet+” tele-collaboration model adopted in Jiangsu and Henan Provinces, and the county-level integrated healthcare alliance model implemented in Shanxi and Zhejiang Provinces (see Table 1 for details).

**Table 1:** Regional practice and exploration of hierarchical diagnosis and treatment system in China.

Model types	Features	Typical regions
The medical consortium collaboration model	A model of establishing cooperation between medical institutions of different levels within a certain area, taking advantage of the concentration of high-quality resources in high-grade medical institutions to play the role of technical radiation and drive to the grassroots.	Beijing, Fujian Province (10–12).
The contracted service model	A model in which health care providers form a long-term stable contractual relationship with residents by signing a continuous service agreement with them as a consortium	The “1+1+1” medical institution combination contracted service in Shanghai; the integrated medical care and nursing smart medical service in Hangzhou (13, 14).
The chronic disease management service model	A model that takes chronic disease management as a breakthrough, and primary care physicians and professional physicians form a team to provide services together	The “chronic disease first, three teachers co-management” service in Xiamen; the “two chronic diseases” whole-cycle health management in Zhejiang Province (15, 16).
The “internet+” tele-collaboration model	An integrated online and offline medical service model based on the Internet and other information technologies to build an integrated medical service model covering pre-consultation, consultation and post-consultation in order to achieve cross-regional, hierarchical, fair and orderly tele-medicine services.	Jiangsu Province, Henan Province (17, 18).
The county-level integrated healthcare alliance model	Guided by county-level hospitals, this model integrates healthcare resources across county and township tiers to form an operationally consolidated consortium, establishing a unified entity that aligns service delivery, accountability, benefit distribution, and governance mechanisms.	Shanxi, Zhejiang Province (19, 20).

#### 2.1.3 Dimension III: service utilization

In an ideal model, medical service provision should satisfy the “positive triangular” structure of the population's healthcare needs, with common illnesses being solved in primary healthcare institutions, while rare and complex diseases with low incidence rates requiring treatment at higher-level institutions (e.g., tertiary hospitals in China). Also, the professionalism of disease diagnosis determines that, in non-emergency cases, the diagnosis of difficult and complicated diseases should firstly rely on primary medical institutions. That is to say, when an individual fall ill, whether or not his condition is serious, and whether or not to go to a high-level medical institution for treatment, does not depend on his own judgement and choice, but rather relies on the diagnosis by primary care practitioners. Therefore, the reasonable path of medical treatment for residents under the hierarchical diagnosis and treatment system is that, 1) In non-emergency cases, residents should first utilize primary healthcare institutions. 2) Only upon diagnosis and confirmation of necessity by primary care practitioners should targeted referrals to higher-level medical institutions occur. In other words, the smooth referral between medical institutions relies on the ‘primary diagnosis' as the gatekeeper. 3) The ‘positive triangle' structure of the population's medical demand determines that the population's utilization rate of primary medical institutions should be higher than that of high-level medical institutions, as a result, the overall utilization of the population's medical services should show a decreasing trend from primary medical institutions to high-level medical institutions.

### 2.2 Data analysis

Based on the above the three-dimensional practice model of China's hierarchical diagnosis and treatment system, this study puts forward 2 basic hypotheses: (1) Primary healthcare institutions should be the first-contact facilities and the principal providers of outpatient services for both urban and rural residents; (2) Secondary-level hospitals, which also are county/district hospitals, should be the main providers of inpatient services for urban and rural residents.

Focusing on the two hypotheses, this study conducts analytical validation using data from the China Health Statistics Yearbook (2008–2023), the Fourth National Health Service Survey Report (2008), the Fifth National Health Service Survey Report (2013), and the Sixth National Health Service Survey Report (2018). Among them, data from the National Health Service Survey Reports are used to analyze the behavioral intention characteristics of residents' choice of first-contact medical institutions. Restricted by the frequency of surveys, these data only cover three time points: 2008, 2013, and 2018. Therefore, this study is unable to conduct a continuous trend analysis of first-contact behavior, and only takes the results of the three surveys as supplementary evidence for phased observation, which are compared with the continuous annual data of outpatient and inpatient services. Data from the China Health Statistics Yearbook are used to analyze the actual utilization scale of outpatient and inpatient services in medical institutions at all levels. With consistent classification criteria for medical institutions, the two types of data are analyzed from the two dimensions of behavioral intention and service utilization respectively which jointly explain the practical characteristics of residents' medical-seeking behavior under the implementation of the hierarchical diagnosis and treatment system. Specific indicators include: the proportion of institutions that are the first point of contact for urban and rural residents suffering from 2-week illnesses; annual outpatient services of medical institutions at different levels, including annual total outpatient visits, proportional distribution, value of structure variation (VSV), degree of structure variation (DSV), and contribution rate of structure variation (CRSV); as well as annual inpatient services of medical institutions at different levels, including annual total inpatient visits, proportional distribution, VSV, DSV, and CRSV. It should be noted that structural variation analysis is a dynamic data processing method for describing trends in overall characteristics and structural changes, and mainly includes three calculation indicators, VSV, DSV, and CRSV. These three indicators are descriptive in nature and do not involve statistical inference, they are only used to describe the magnitude and contribution of structural changes. Among them, VSV is defined as the difference between the proportional composition of each component at the end and the start of a period, reflecting the magnitude and direction of changes in the proportional composition of each component during the period. The formula is: *VSV* = *X*_*i*1_−*X*_*i*0_, where Xi represents the proportional composition of each component, with 0 denoting the initial period and 1 the final period. A negative VSV indicates a downward trend, while a positive value signifies an upward trend (8). DSV is calculated as the sum of the absolute differences between the proportional compositions at the start and end of the period, reflecting the total magnitude of structural changes across all components. The formula is: *DSV* = ∑|*X*_*i*1_− *X*_*i*0_| . A higher DSV value indicates greater overall structural variation. CRSV measures the proportion of the absolute VSV of each component relative to the total DSV, quantifying the impact of individual components on the overall structural change (9). It should be noted that some results approaching 50% reflect inherent features of the calculation method, and do not mean that a certain type of institution contributed half of the structural change or occupied an absolutely primary position. Results should be interpreted in conjunction with the direction and magnitude of the VSV. The formula is: $|X_{i1}-X_{i0}|/DSV^{*}100\%.$

To further observe the impact of key policy milestones, this study divides the full study period 2007–2022 into three stages—2007–2015, 2016–2019, and 2020–2022—with the full implementation of the hierarchical diagnosis and treatment system in 2015 and the onset of COVID-19 in 2020 as the dividing points for comparative analysis. Meanwhile, to test the robustness of the results, this study carries out a sensitivity analysis by excluding the pandemic period (2020–2022) and recalculating the average development rates for all levels of medical institutions over 2007–2019. These estimates are then compared with the full-period results to exclude potential interference from the pandemic on long-term structural trends. Notably, this study is a macro-level descriptive trend analysis, designed to reveal the changing patterns of service utilization among medical institutions at different levels in China from 2007 to 2022. Given the observational nature of the data, this study cannot identify the net effects of hierarchical diagnosis and treatment policies nor establish causal relationships. The observed trends may be associated with multiple factors other than policy interventions, including demographic shifts, economic development, and technological progress.

## 3 Results

### 3.1 Structural distribution of first-contact institutions for 2-week illnesses among urban and rural residents

**Figure 2:**
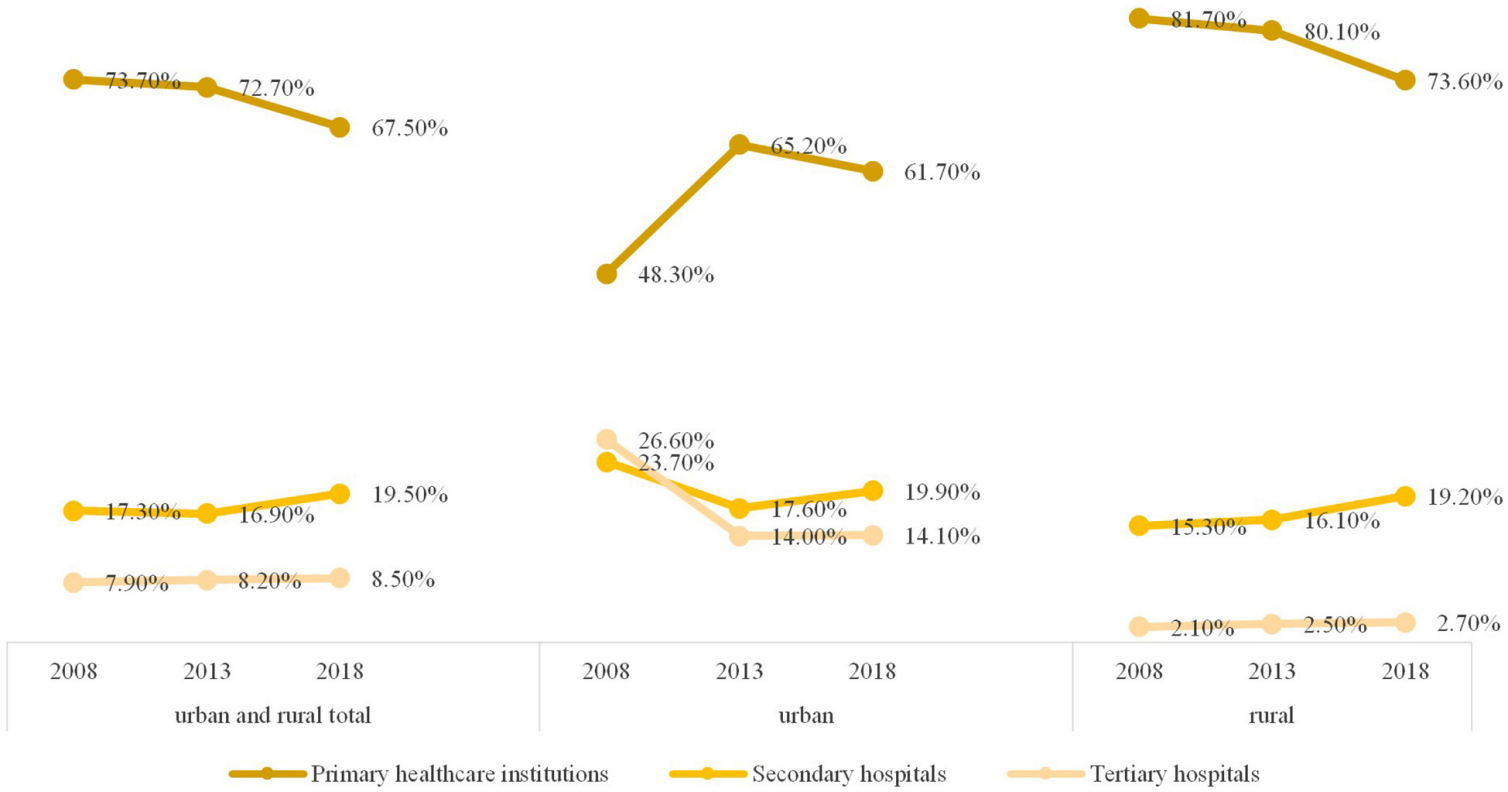
Composition of first-contact institutions for 2-week illnesses among urban and rural residents.

According to the results of the most recent national health service survey (the sixth national health service survey) published by China, as shown in Figure 2, it can be seen that the proportion of urban and rural residents choosing primary healthcare institutions for the first consultation of a 2-week illness is 67.50%, with higher rates in rural areas (73.60%) than urban areas (61.70%). The proportion of residents choosing secondary hospitals in the county and district for their first visit was 19.50%, slightly higher in urban areas (19.90 %) than in rural areas (19.20 %). Eight point five zero percent of the residents still chose a tertiary hospital for their first visit, and this was notably higher in urban areas (14.10%) than in rural areas (2.70%).

Based on the results of the fourth, fifth and sixth national health service in China, in 2008, 2013, and 2018, the proportion of primary healthcare institutions providing first-visit services for 2-week illnesses was 73.70, 72.70, and 67.50%, respectively showing a declining trend. Among them, the proportion of urban residents choosing primary healthcare institutions for their first consultation in the three surveys was 48.30, 65.20, and 61.70%, respectively showing a rise-first-then-decline pattern; the proportion of rural residents was 81.70, 80.10, and 73.60%, respectively showing a declining trend. In contrast, the proportion of urban and rural residents choosing secondary hospitals at the county and district levels for their first consultation increased, with the three survey results being 17.30, 16.90, and 19.50%, respectively. This upward trend was more obvious among rural residents, whose proportions were 15.30, 16.10, and 19.20%, respectively while urban residents displayed a trend of falling first and then rising, with proportions of 23.70, 17.60, and 19.90%, respectively. The proportion of urban and rural residents choosing tertiary hospitals remained around 8.0%, and the proportions for urban residents were 26.60, 14.00, and 14.10%, respectively.

### 3.2 Utilization of outpatient services at different levels of medical institutions among urban and rural residents

#### 3.2.1 Annual outpatient service provision across different levels of medical institutions

As shown in Table 2, from 2007 to 2022, the annual outpatient visits handled by primary healthcare institutions increased from 3,084.09 million to 4,480.77 million, with an average annual growth rate of 102.62%; the number of outpatient visits undertaken by secondary hospitals increased from 744.75 million to 1,203.75 million, with an average development rate of 103.25%; and the number of outpatient visits undertaken by tertiary hospitals increased from 553.89 million to 2,228.55 million, with an average development rate of 109.73%. For the different levels of institutions, primary healthcare institutions maintained the highest absolute volume of outpatient services, while tertiary hospitals demonstrated the strongest growth momentum in outpatient services. To eliminate the influence of population size changes, Table 2 also presents the average number of outpatient visits per institution by level. From 2007 to 2022, the average number of outpatient visits in primary healthcare institutions increased from 3,491 to 4,514, and in tertiary hospitals from 468,606 to 632,572. By stages, the trend in average outpatient visits was generally consistent with that in the aggregate analysis (see Table 2).

**Table 2:** Annual outpatient service provision across different level of medical institutions.

Year	Primary healthcare institutions			Secondary hospitals			Tertiary hospitals		
	Total outpatient visits (10,000 people)	Average number of outpatient visits (per institution)	Sequential growth rate %	Total outpatient visits (10,000 people)	Average number of outpatient visits (per institution)	Sequential growth rate %	Total outpatient visits (10,000 people)	Average number of outpatient visits (per institution)	Sequential growth rate %
2007	308,409	3,491	/	74,475	112,704	/	55,389	468,606	/
2008	311,842	3,613	101.11	83,021	122,449	111.47	62,128	521,206	112.17
2009	354,232	3,992	113.59	88,840	136,195	107.01	68,939	559,118	110.96
2010	375,729	4,143	106.07	93,120	143,882	104.82	76,046	592,261	110.31
2011	395,896	4,286	105.37	99,199	153,368	106.53	89,808	641,943	118.10
2012	431,000	4,692	108.87	105,000	160,159	105.85	109,000	671,182	121.37
2013	450,049	4,882	104.42	109,169	162,720	103.97	123,822	692,904	113.60
2014	454,873	4,921	101.07	114,709	167,458	105.07	139,804	715,478	112.91
2015	454,761	4,892	99.98	117,233	156,436	102.20	149,765	705,439	107.12
2016	458,454	4,899	100.81	121,667	153,155	103.78	162,785	729,323	108.69
2017	465,109	4,932	101.45	126,785	150,540	104.21	172,643	737,788	106.06
2018	463,096	4,852	99.57	128,493	142,501	101.35	185,479	727,938	107.44
2019	476,052	4,930	102.80	134,343	138,684	104.55	205,701	748,276	110.90
2020	431,840	4,406	90.71	115,607	111,118	86.05	179,825	600,217	87.42
2021	446,673	4,510	103.43	125,453	115,646	108.52	223,144	681,356	124.09
2022	448,077	4,514	100.31	120,375	108,008	95.95	222,855	632,572	99.87
Average growth rate	/	/	102.62	/	/	103.25	/	/	109.73

As shown in Table 3, by stages, from 2007 to 2015, the average annual growth rate of outpatient visits in primary healthcare institutions was 104.97%, 101.26% during 2016–2019 and 101.86% during 2020–2022. In 2020, affected by the COVID-19 pandemic, outpatient visits in primary healthcare institutions decreased, from 4,760.52 million in 2019 to 4,318.40 million in 2020, and recovered gradually after 2021. For secondary hospitals, the average annual growth rates were 105.84% during 2007–2015, 103.36% during 2016–2019, and 102.04% during 2020–2022. For tertiary hospitals, the average annual growth rates were 113.24% during 2007–2015, 108.11% during 2016–2019, and 111.32% during 2020–2022. Similarly, tertiary hospitals also saw a temporary decline in outpatient visits in 2020.

**Table 3:** Average growth rate of outpatient services in different levels of medical institutions by stages (%).

Year	Primary healthcare institutions	Secondary hospitals	Tertiary hospitals
2007–2015	104.97	105.84	113.24
2016–2019	101.26	103.36	108.11
2020–2022	101.86	102.04	111.32

Considering the potential interference of the COVID-19 pandemic on the long-term trend of outpatient service utilization, this study further conducted a sensitivity analysis. After excluding the pandemic-affected years, the average development rates of primary healthcare institutions, secondary hospitals, and tertiary hospitals from 2007 to 2019 were 103.68, 105.04, and 111.55%, respectively higher than the full-sample rates of 102.62%, 103.25%, and 109.73%. Although the growth rates of all levels increased, tertiary hospitals still achieved the fastest growth, followed by secondary hospitals, and primary healthcare institutions the slowest.

#### 3.2.2 Structural variation analysis in outpatient service provision across different levels of medical institutions

In terms of the structural variations in outpatient service provision across different levels of medical institutions, as shown in Table 4, for primary healthcare institutions, VSV of the number of outpatient visits was negative except for 2009, 2020 and 2022, indicating an overall downward trend and CRSV exceeded 40% in most years, with exceptions in 2010, 2012, and 2013. For secondary hospitals at county and district level, VSV of the number of outpatient visits was negative except for 2008 and 2014–2017, while CRSV was relatively low in all years (below 30% in all years except 2009 and 2022). For tertiary hospitals, VSV of the number of outpatient visits was positive except for 2009 and 2020, reflecting an upward trajectory, and CRSV was greater than 30% in all years except 2008, 2009, 2020 and 2022.

**Table 4:** Structural variation analysis of outpatient service provision across different levels of medical institutions.

Year	VSV			DSV (%)	CRSV (%)		
	Primary healthcare institutions	Secondary hospitals	Tertiary hospitals		Primary healthcare institutions	Secondary hospitals	Tertiary hospitals
2008	−0.0213	0.0117	0.0096	4.2621%	50.0000%	27.5462%	22.4538%
2009	0.0095	−0.0082	−0.0013	1.8927%	50.0000%	43.0968%	6.9032%
2010	−0.0023	−0.0026	0.0049	0.9834%	23.3937%	26.6063%	50.0000%
2011	−0.0127	−0.0013	0.0140	2.7965%	45.3648%	4.6352%	50.0000%
2012	−0.0086	−0.0068	0.0154	3.0898%	27.9648%	22.0352%	50.0000%
2013	−0.0093	−0.0030	0.0123	2.4577%	37.9463%	12.0537%	50.0000%
2014	−0.0177	0.0019	0.0158	3.5341%	50.0000%	5.2997%	44.7003%
2015	−0.0111	0.0007	0.0104	2.2295%	50.0000%	3.2556%	46.7444%
2016	−0.0130	0.0013	0.0116	2.5927%	50.0000%	5.1841%	44.8159%
2017	−0.0088	0.0021	0.0067	1.7511%	50.0000%	11.7722%	38.2278%
2018	−0.0124	−0.0005	0.0129	2.5754%	48.1516%	1.8484%	50.0000%
2019	−0.0126	−0.0007	0.0134	2.6729%	47.2318%	2.7682%	50.0000%
2020	0.0105	−0.0057	−0.0048	2.0904%	50.0000%	27.0608%	22.9392%
2021	−0.0321	−0.0012	0.0333	6.6659%	48.1835%	1.8165%	50.0000%
2022	0.0046	−0.0056	0.0010	1.1254%	40.7590%	50.0000%	9.2410%

By stages, from 2008 to 2015, the VSV of primary healthcare institutions was negative except for 2009, with CRSV exceeding 40% in most years; from 2016 to 2019, VSV remained consistently negative, indicating a downward trend, with CRSV maintained between 47% and 50%; from 2020 to 2022, VSV showed obvious fluctuations. For secondary hospitals, VSV was mostly negative during 2008–2015, with CRSV generally below 30%; during 2016–2019, VSV turned from positive to negative, with CRSV still below 20%; during 2020–2022, VSV was negative throughout, with CRSV reaching 50% in 2022 but remaining low in other years. For tertiary hospitals, VSV was positive during 2008–2015 except in 2009, with CRSV mostly above 30%; during 2016–2019, VSV remained positive, with CRSV declining somewhat in 2016–2018; during 2020–2022, VSV was negative in 2020, turned positive with CRSV reaching 50% in 2021, and remained positive in 2022 with a lower CRSV.

**Figure 3:**
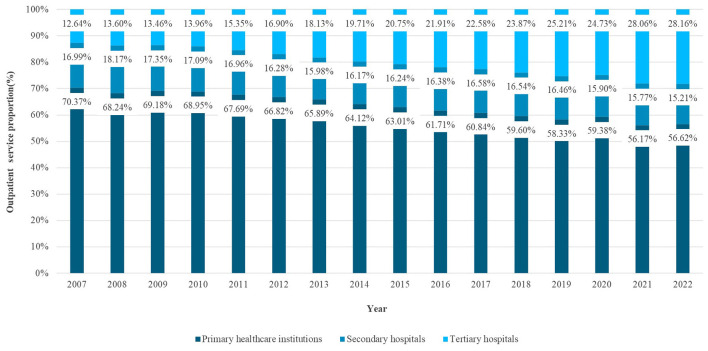
The proportion of outpatient service provision across different levels of medical institutions (2007–2022).

Figure 3 shows the distribution of outpatient service provision across different levels of medical institutions from 2007 to 2022. Combined with the results of structural variations analysis, it can be concluded that primary healthcare institutions exhibited a progressive decline in their share of outpatient services with substantial contraction over the period; for secondary hospitals at county and district level, the proportion of outpatient service has shown a declining (2009–2013) - rising (2014- (2017)-then decline (2018–2022) trend, though with limited overall variation; and conversely, tertiary hospitals displayed consistent expansion in outpatient service share. By the end of 2022, primary healthcare institutions were responsible for about 56.62% of outpatient services, secondary hospitals for about 15.21%, and tertiary hospitals for about 28.16%.

By stages, from 2007 to 2015, the proportion of outpatient services provided by primary healthcare institutions declined from 70.37% to 63.01%, representing a relatively obvious decrease; from 2016 to 2019, the decline moderated, with the proportion falling from 61.71% to 58.33%; during 2020–2022, affected by the COVID-19 pandemic, the proportion saw short-term fluctuations. For secondary hospitals, the proportion fluctuated slightly during 2007–2015; from 2016 to 2019, it rebounded modestly; during 2020–2022, it continued to decline, from 15.90% to 15.21%. For tertiary hospitals, the proportion increased steadily during 2007–2015, rising from 12.64% to 20.75%; from 2016 to 2019, it further increased to 25.21%; during 2020–2022, it continued to rise, with the overall service structure still characterized by upward concentration.

### 3.3 Utilization of inpatient services at different levels of medical institutions among urban and rural residents

#### 3.3.1 Annual inpatient service provision across different levels of medical institutions

As shown in Table 5, from 2007 to 2022, the annual inpatient visits handled by primary healthcare institutions increased from 31.51 million to 47.25 million, with an average development rate of 102.74%; the number of annual inpatient services undertaken by secondary hospitals increased from 34.76 million to 65.21 million, with an average development rate of 104.28%; and the number of annual inpatient services undertaken by tertiary hospitals increased from 20.34 million increased to 116.35 million, with an average development rate of 112.33%. Among the different levels of medical institutions, tertiary hospitals had the highest average rate of development in terms of the number of annual inpatient visits. Since 2016, the total number of annual inpatient visits in tertiary hospitals has exceeded that in secondary hospitals, making them the main providers of inpatient care. To eliminate the influence of population size changes, Table 5 also presents the average number of inpatient visits per institution at each level. From 2007 to 2022, the average number of inpatient visits in primary healthcare institutions decreased from 571 to 568, and in tertiary hospitals increased from 17,206 to 33,026. By stages, the trends in average inpatient visits were generally consistent with the aggregate analysis (see Table 5).

**Table 5:** Annual inpatient service provision across different levels of medical institutions.

Year	Primary healthcare institutions			Secondary hospitals			Tertiary hospitals		
	Total inpatient admissions (10,000 people)	Average inpatient admissions (per institution)	Sequential growth rate %	Total inpatient admissions (10,000 people)	Average inpatient admissions (per institution)	Sequential growth rate %	Total inpatient admissions (10,000 people)	Average inpatient admission (per institution)	Sequential growth rate %
2007	3,151	—	/	3,476	5,261	/	2,034	17,206	/
2008	3,900	571	123.77	4,061	5,990	116.83	2,327	19,520	114.41
2009	4,543	641	116.49	4,636	7,107	114.16	2,668	21,641	114.65
2010	4,414	582	97.16	5,116	7,904	110.35	3,097	24,118	116.08
2011	4,311	569	97.67	5,567	8,608	108.82	3,717	26,571	120.02
2012	4,903	640	113.73	6,242	9,521	112.13	4,726	29,101	127.15
2013	5,029	649	102.57	6,621	9,869	106.07	5,450	30,499	115.32
2014	4,892	626	97.27	7,006	10,227	105.81	6,291	32,195	115.43
2015	5,001	626	102.23	7,121	9,503	101.65	6,828	32,162	108.54
2016	5,204	647	104.06	7,570	9,530	106.31	7,686	34,436	112.57
2017	5,619	692	107.97	8,006	9,506	105.75	8,396	35,882	109.24
2018	5,586	679	99.41	8,177	9,068	102.13	9,292	36,469	110.67
2019	5,446	661	97.50	8,380	8,651	102.49	10,483	38,134	112.82
2020	4,824	579	88.58	6,965	6,695	83.11	9,373	31,285	89.41
2021	4,712	563	97.68	6,890	6,351	98.92	11,252	34,357	120.05
2022	4,725	568	100.28	6,521	5,851	94.64	11,635	33,026	103.40
Average growth rate	/	/	102.74	/	/	104.28	/	/	112.33

As shown in Table 6, by stages, from 2007 to 2015, the average annual growth rate of inpatient visits in primary healthcare institutions was 105.94%, 101.53% during 2016–2019 and 98.97% during 2020–2022. In 2020, affected by the COVID-19 pandemic, inpatient service utilization in primary healthcare institutions decreased, from 54.46 million in 2019 to 48.24 million in 2020, recovering gradually after 2021. For secondary hospitals, the average annual growth rates were 109.38% during 2007–2015, 103.45% during 2016–2019, and 96.76% during 2020–2022. For tertiary hospitals, the average annual growth rates were 116.34% during 2007–2015, 110.90% during 2016–2019, and 111.42% during 2020–2022. Similarly, tertiary hospitals also experienced a temporary decline in inpatient visits in 2020, with a comparatively faster recovery.

**Table 6:** Average growth rate of inpatient services in different levels of medical institutions by stages (%).

Year	Primary healthcare institutions	Secondary hospitals	Tertiary hospitals
2007–2015	105.94	109.38	116.34
2016–2019	101.53	103.45	110.90
2020–2022	98.97	96.76	111.42

Similarly, considering the potential interference of the COVID-19 pandemic on the long-term trend of inpatient service utilization, the sensitivity analysis shows that the results for inpatient services were consistent with those for outpatient services. After excluding the pandemic-affected years, the average development rates of primary healthcare institutions, secondary hospitals, and tertiary hospitals over 2007–2019 were 104.67, 107.61, and 114.64%, respectively all higher than the full-period levels, while the hierarchical growth pattern remained unchanged.

#### 3.3.2 Structural variation analysis in inpatient service provision across different levels of medical institutions

Regarding the structural variations in inpatient service provision across different levels of medical institutions, as shown in Table 7, for primary healthcare institutions, VSV of the number of inpatient visits was positive in 2008–2009, with CRSV reaching 50%; and was negative in 2010–2019, with CRSV of more than 15% except for 2017; and it is of concern that, possibly due to COVID-19 pandemic, VSV in 2020 was positive, and then fluctuated in 2021–2022, while CRSV was relatively lower than other institutions. For secondary hospitals at county and district level, VSV of the number of inpatient visits has been negative since 2010, while CRSV was relatively low in all years (below 15% in all years except 2011 and 2014). In contrast, for tertiary hospitals, VSV of the number of inpatient visits has been positive since 2010, and CRSV was greater than 35% in all years.

**Table 7:** Structural variation analysis of inpatient service provision across different levels of medical institutions.

Year	VSV			DSV (%)	CRSV (%)		
	Primary healthcare institutions	Secondary hospitals	Tertiary hospitals		Primary healthcare institutions	Secondary hospitals	Tertiary hospitals
2008	0.0153	−0.0066	−0.0087	3.0535%	50.0000%	21.6393%	28.3607%
2009	0.0044	−0.0034	−0.0010	0.8780%	50.0000%	38.8256%	11.1744%
2010	−0.0339	0.0138	0.0201	6.7808%	50.0000%	20.4117%	29.5883%
2011	−0.0325	0.0043	0.0281	6.4933%	50.0000%	6.6611%	43.3389%
2012	−0.0082	−0.0162	0.0244	4.8733%	16.7723%	33.2277%	50.0000%
2013	−0.0148	−0.0061	0.0209	4.1880%	35.4025%	14.5975%	50.0000%
2014	−0.0251	−0.0020	0.0272	5.4317%	46.2905%	3.7095%	50.0000%
2015	−0.0050	−0.0094	0.0144	2.8870%	17.4861%	32.5139%	50.0000%
2016	−0.0096	−0.0058	0.0153	3.0692%	31.1332%	18.8668%	50.0000%
2017	0.0008	−0.0064	0.0056	1.2875%	6.2627%	50.0000%	43.7373%
2018	−0.0129	−0.0089	0.0218	4.3539%	29.5971%	20.4029%	50.0000%
2019	−0.0182	−0.0099	0.0282	5.6368%	32.3632%	17.6368%	50.0000%
2020	0.0039	−0.0156	0.0117	3.1201%	12.5749%	50.0000%	37.4251%
2021	−0.0218	−0.0276	0.0494	9.8852%	22.0303%	27.9697%	50.0000%
2022	0.0003	−0.0165	0.0162	3.2965%	0.9855%	50.0000%	49.0145%

By stages, from 2008 to 2015, the VSV of primary healthcare institutions was negative except in 2008 and 2009, with CRSV exceeding 30% in most years; from 2016 to 2019, VSV was predominantly negative, and CRSV declined somewhat; from 2020 to 2022, VSV fluctuated notably, being positive in 2020 and 2022 but negative in 2021. For secondary hospitals, VSV was mostly negative during 2008–2015, with CRSV generally below 30%; during 2016–2019, VSV remained consistently negative, with CRSV reaching 50% in 2017 but staying low in other years; during 2020–2022, VSV was negative throughout, with CRSV rising to 50% in 2022. For tertiary hospitals, VSV was positive during 2008–2015 except in 2008 and 2009, with CRSV mostly above 25%; during 2016–2019, VSV remained consistently positive, and CRSV reached 50% in 2016, 2017, and 2019; during 2020–2022, VSV remained positive, with CRSV rising to 49.01% in 2022, indicating a sustained upward trend.

**Figure 4:**
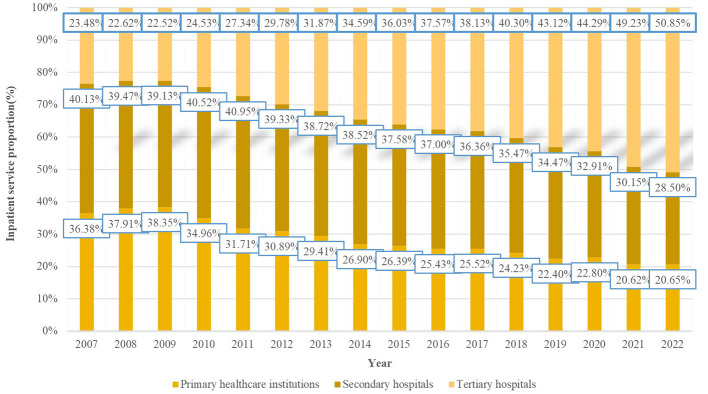
The proportion of inpatient service provision across different levels of medical institutions (2007–2022).

Figure 4 shows the proportion of inpatient services provided by different levels of medical institutions from 2007 to 2022. Combined with the results of structural variations analysis, it can be concluded that the proportion of inpatient services provided by primary healthcare institutions and secondary hospitals at county and district levels has been gradually declining, with a greater rate of decline, whereas the proportion of inpatient services provided by tertiary hospitals has been gradually increasing, with a greater rate of increase. This trend has led to tertiary hospitals becoming the main providers of inpatient services, with their share of inpatient services exceeding 50% by 2022.

By stages, from 2007 to 2015, the proportion of inpatient services provided by primary healthcare institutions declined from 36.38% to 26.39%, representing a fluctuating decrease; from 2016 to 2019, the proportion continued to decline, from 25.43% to 22.40%; during 2020–2022, the overall trend leveled off. For secondary hospitals, the proportion declined from 40.13% to 37.58% during 2007–2015, with fluctuations; from 2016 to 2019, the downward trend accelerated; during 2020–2022, the proportion continued to decline, from 32.91% to 28.50%. For tertiary hospitals, the proportion increased steadily from 23.48% to 36.03% during 2007–2015; from 2016 to 2019, it continued to rise; during 2020–2022, it further increased from 49.23% to 50.85%, with the trend of inpatient resources concentrating in tertiary hospitals continuing to strengthen.

## 4 Discussion

The implementation of the hierarchical diagnosis and treatment system is a gradual deepening process. During 2007–2015, policies focused on system construction and pathway exploration, aiming to build a healthcare service system covering both urban and rural areas, guide general care downward to the grassroots level, and gradually implement pilot programs for community first-contact and two-way referrals, thereby promoting the initial formation of the hierarchical diagnosis and treatment system. During 2016–2019, policy objectives were further clarified: by 2017, the proportion of consultations at primary healthcare institutions was expected to increase notably; by 2020, a hierarchical diagnosis and treatment model featuring “primary care first contact, two-way referrals, separate management of acute and chronic conditions, and upward and downward linkages” was to be basically established.

Combined with the empirical results of this study, it can be observed that there is a certain asynchrony between the evolution of policy objectives and the actual changes in healthcare-seeking patterns. During 2007–2015, although the primary healthcare service system was continuously improved, the growth rate of outpatient services in primary healthcare institutions remained lower than that in tertiary hospitals, and the proportion of primary care first-contact visits showed a declining trend. During 2016–2019, against the backdrop of comprehensive policy promotion, the growth rates of tertiary hospitals still exceeded those of primary and secondary institutions, and the trend of patient concentration in tertiary hospitals remained pronounced. These policy context and empirical insights inform the further discussion below.

### 4.1 The order of medical treatment in China, based on hierarchical diagnosis and treatment system, still needs to be further strengthened

As outlined earlier, establishing a rational and well-organized medical care order helps improve the utilization efficiency of health resources and is integral to optimizing health system performance. Through analyzing the operational dynamics of China's hierarchical medical institutions in healthcare delivery, our analysis confirms that, on one hand, from the perspective of the overall trend of residents' healthcare-seeking intentions and actual service utilization, primary healthcare institutions currently serve as the primary channel for both initial medical consultations (accounting for 67.5% of first-contact cases in the sixth national health service survey) and routine outpatient services (representing 56.7% of total visits in 2022), and secondary hospitals also handle a considerable volume of inpatient services, both demonstrating an effectively tiered healthcare delivery system. This is closely tied to the structural design of China's healthcare system, which establishes clear functional roles for different medical institutions across distinct tiers of care, thereby laying a solid foundation for orderly patient referrals. On the other hand, combined with changes in residents' willingness to seek initial care at primary facilities and dynamic shifts in service provision among medical institutions at different levels, a noticeable gap still exists between the current orderly healthcare-seeking behavior and the vision of “first-contact care at primary institutions and two-way referrals” advocated by the hierarchical diagnosis and treatment system. From the perspective of first-visit services, the proportion of urban and rural residents choosing primary care institutions for first-contact care has declined to varying degrees, with a more notable decrease among rural residents. This urban-rural disparity may be closely related to practical factors such as the orientation of health insurance policies and the quality of medical services. Studies have shown that the health insurance reimbursement rate is an important factor influencing residents' primary care first-contact behavior, and that increasing the reimbursement rate for primary care helps guide residents to seek care at the grassroots level (21). Moreover, changes in health insurance reimbursement rates have a greater impact on the healthcare-seeking behavior of rural residents and low-income groups (22). Studies have also shown that physical accessibility is an important factor influencing rural residents' willingness to seek care (23). Compared with urban areas, medical resources in rural areas are relatively sparse, and residents often need to spend more time and transportation costs to seek medical care. Under the combined influence of the above factors, the first-contact care behavior of urban and rural residents presents different patterns of change; From the perspective of outpatient and inpatient services, primary healthcare facilities have shown an overall negative trend in outpatient visits, while secondary hospitals have consistently recorded negative structural change values in inpatient service provision since 2010, reflecting a decline in the relative share of primary and secondary hospitals in the service structure. Combined with absolute service volume (see Tables 2 and 5), the actual service volume of primary and secondary hospitals continued to grow, indicating that this change is, to some extent, a relative adjustment brought about by the rapid expansion of tertiary hospitals. The reasons may be multifaceted, including both the insufficiently manifested effects of policy implementation and factors such as patients' healthcare-seeking inertia and the resource aggregation effect of tertiary hospitals. Fundamentally, this phenomenon needs to be understood from both the demand side and the supply side. From the demand side, it remains attributable to patients' persistent quality-oriented preferences in healthcare-seeking behavior. Peer-reviewed evidence confirms that therapeutic efficacy dominates healthcare choices, yet information asymmetries prevent quality discernment. When combined with Veblen-good characteristics of medical demand and technology fetishism, these factors generate systematic deviations from value-based care selection (24, 25). From the supply side, primary healthcare institutions face challenges such as insufficient talent and high physician turnover, making it difficult to fully meet residents' expectations for common disease diagnosis and chronic disease management. Secondary hospitals also lag behind higher-level institutions in specialized capabilities and technical equipment. These factors jointly drive residents to flow toward higher-level medical institutions, consequently leading to a relative decline in the service share of primary and secondary institutions. Based on the above analysis, it should be noted that the hypothesis that “secondary-level hospitals at the county/district level should be the main providers of inpatient services for urban and rural residents” is a developmental goal based on the ideal division of labor structure of hierarchical diagnosis and treatment. From the actual trend, current inpatient services are still dominated by tertiary hospitals, and secondary hospitals have not yet become the main inpatient service providers, indicating a gap between the actual situation and the ideal goal. This gap suggests that shifting the focus of inpatient services downward and building an inpatient service system with secondary hospitals as important pillars remains a direction for future reform. Under the current resource distribution and disease structure characteristics, the realization of this goal still needs to be gradually advanced through division of labor optimization and capacity improvement.

### 4.2 China's policies to promote the hierarchical diagnosis and treatment system still require broader implementation to achieve full coverage

As previously stated, in China, under the national policy guidance since 2015 to “establish a scientifically sound healthcare-seeking order and gradually develop a hierarchical diagnosis and treatment system”, regions including Beijing, Shanghai, Xiamen, Guangdong, Zhejiang, and Shanxi have actively explored mechanisms to create efficient, responsibility-defined collaborative networks among healthcare institutions at all levels. From the perspective of practice types, various regions have mainly developed different policy models such as medical consortium collaboration, contracted services, “Internet+” tele-collaboration, and county-level integrated healthcare alliances, all of which aim to optimize the division of labor among different levels of medical institutions and guide patients to seek medical care in an orderly manner as the reform direction. Combined with the empirical results of this study, the utilization of healthcare services at the national level still shows a trend of concentration in tertiary hospitals, and the service capacity and diversion role of primary healthcare institutions and secondary hospitals still need to be improved. This also means that, at the current stage, various policies have not yet formed systematic and observable universal effects at the national level. In comparison, policy models such as county-level integrated healthcare alliances, which emphasize regional resource integration and functional division of labor, are more aligned with the practical needs of hierarchical diagnosis and treatment reflected in this study in terms of reform direction. However, their actual effects cannot be accurately verified in the macro data of this study. However, it should be noted that these policy implementations demonstrate distinct regional characteristics, with the changes brought about by these policies consequently confined to specific geographical contexts. For instance, Zheng Caiyun et al. (26) demonstrated through Rank Sum Ratio (RSR) analysis of Guangdong's county-level medical consortiums, preliminary outcomes have emerged across three key indicators: the proportion of primary care visits, bed utilization rates at grassroots institutions, and the share of Grade III-IV surgeries performed at tertiary hub hospitals. Employing Data Envelopment Analysis (DEA), Jiang Tianyu et al. (27) and Feng Mingyu et al. (28), respectively evaluated the service efficiency of township health centers and hub hospitals within county-level medical consortiums. Their findings indicated that in most pilot regions, the technical efficiency (TE) and pure technical efficiency (PTE) indices of county hospitals surpassed the national average for public county hospitals, while township health centers also demonstrated significant improvements in scale efficiency (SE). Building on existing research, it can be concluded that China's regional explorations of hierarchical diagnosis and treatment systems have played a positive role in optimizing resource allocation and service provision across different levels of medical institutions within local contexts. The next step should focus on scaling up these proven policies to broader national implementation, thereby expanding the scope of policy application to further promote the formation of an orderly healthcare-seeking order in China.

### 4.3 The role of tertiary hospitals in China's hierarchical diagnosis and treatment system requires further optimization

Within China's healthcare delivery system, tertiary hospitals serve dual functions: aggregating premium medical resources while maintaining their mandated role in providing diagnosis and treatment for complex and critical diseases. In this context, China's national policy documents in recent years—including the *Guiding Opinions of General Office of the State Council of the People's Republic of China on Promoting the Construction of the Hierarchical Diagnosis and Treatment System* [State Council Document (2015) No. 70], *Opinions of the General Office of the State Council of the People's Republic of China on Strengthening Performance Assessment in Tertiary Public Hospitals* [State Council Document (2019) No. 4], *Opinions of the General Office of the State Council of the People's Republic of China on Promoting High-Quality Development of Public Hospitals* [State Council Document (2021) No. 18], *Tertiary Hospital Accreditation Standards (2022 Edition)* [NHC Medical Administration Document (2022) No. 31]—have consistently reinforced the role of tertiary hospitals in providing diagnosis and treatment for complex and critical diseases. However, based on the inherent information asymmetry in healthcare markets, the concentration of premium medical resources in tertiary hospitals has also attracted a large number of non-emergency patients to seek care at higher-level institutions. This phenomenon aligns with the characteristics of the “siphon effect” exerted by tertiary hospitals on medical resources and patients. It should be noted that this pattern is not driven by a single factor, but is the result of multiple factors working together—including supply-side forces such as hospital expansion and technological concentration, as well as demand-side factors such as patient preferences and trust in higher-level institutions. These factors jointly drive patient concentration in tertiary hospitals, which to a considerable extent, has undermined the role of tertiary hospitals in providing diagnosis and treatment for complex and critical diseases, and has also led to resource idleness and operational inefficiency in secondary and lower-level medical institutions. As evidenced by this study's findings—consistent with prior research (29, 30) —the concentration of healthcare-seeking behavior toward tertiary hospitals remains notable among Chinese residents, particularly for inpatient services. This phenomenon is the result of the combined effects of demand-side and supply-side factors. From the demand side, population aging and changes in disease patterns have increased the demand for inpatient services, and patients' pursuit of high-quality medical services leads them to prioritize tertiary hospitals with better technology and higher reputation in their healthcare choices. From the supply side, the continuous expansion of beds in tertiary hospitals and the concentration of advanced diagnostic and treatment technologies have strengthened their capacity to receive a large number of patients. These factors jointly drive patients to continuously concentrate in tertiary hospitals. Therefore, this study concludes that, in promoting the decentralization of healthcare resources in China, it is imperative to reassess and optimize the role of tertiary hospitals within the hierarchical diagnosis and treatment system. This requires transforming the traditional fragmented care model by fostering integrated delivery networks between tertiary hospitals, secondary hospitals, and primary healthcare institutions. Such systems should feature: end-to-end clinical collaboration pathways; Value-based payment mechanisms (e.g. episode-based bundled payments, population-based capitation) to incentivize appropriate division of labor and effective coordination across all levels of medical facilities (31).

## 5 Conclusion

Through systematic analysis of the tiered healthcare system and empirical tracking of patient flows in China, this study reveals persistent gaps in establishing well-ordered healthcare-seeking patterns. These findings highlight two critical imperatives: (1) although various regions have developed diverse policy explorations in models such as medical alliances and contracted services, the relevant practices remain largely confined to specific regions and have not yet achieved systematic and observable universal effectiveness at the national level. Therefore, expanding the implementation scope of existing hierarchical diagnosis and treatment policies, and (2) with the varying degrees of decline in the proportion of urban and rural residents choosing primary healthcare institutions for first-contact care, the continuous decline in the relative shares of outpatient and inpatient services in primary and secondary hospitals, and the still notable trend of patient concentration in tertiary hospitals, developing integrated healthcare delivery systems with vertically coordinated services and horizontally regulated competition among medical institutions at all levels should be a critical future direction for China's health system reform.

### 5.1 Limitations

This study used per capita service volume to eliminate the influence of population size changes. However, due to data limitations, it was unable to further control for changes in population age structure and disease spectrum and could not fully isolate the impact of population composition on healthcare demand. This may affect the comprehensiveness of assessments regarding the role of the health system itself.

### 5.2 Future research prospects

This study focuses on the categorization and description of service provision, policy practice and service utilization. Future research may further analyze the causal pathways and feedback mechanisms among the three, so as to deepen the understanding of the operational mechanism of the hierarchical diagnosis and treatment system. Meanwhile, further mechanism verification can be carried out based on the analytical framework of this study to identify the different roles of tertiary hospital resource expansion and primary care capacity improvement in optimizing the healthcare-seeking order, and explore their relative importance in driving the downward flow or upward concentration of patients. Additionally, future research could further analyze the relative contributions of demographic changes and health system reforms to healthcare-seeking patterns, based on more detailed population and disease surveillance data.

## Data Availability

The original contributions presented in the study are included in the article/Supplementary material, further inquiries can be directed to the corresponding author.
