# Supplementary material for: How to promote orderly access to medical care: an empirical analysis based on the Chinese experience
Source: Front Public Health. 2026 Apr 2;14:1766341. doi: 10.3389/fpubh.2026.1766341 (PMC13083061; doi:10.3389/fpubh.2026.1766341)
Supplement: Supplementary file 1 [file Table_1.docx]

**Appendix:**

**Table A1-1 Structural Variation Analysis of Outpatient Service Provision of Different Types of Primary Healthcare Institutions**

| **Year** | **VSV** | | | | | **CRSV (%)** | | | | | **DSV（%）** |
| --- | --- | --- | --- | --- | --- | --- | --- | --- | --- | --- | --- |
|  | **First-level hospitals** | **Community health service centers (stations)** | **Township health centers** | **Village clinics** | **Other**  **primary healthcare institutions** | **First-level hospitals** | **Community health service centers (stations)** | **Township health centers** | **Village clinics** | **Other**  **primary healthcare institutions** |  |
| 2008 | 0.0014 | 0.0046 | 0.0078 | -0.0169 | -0.0183 | 1.9346% | 6.5991% | 11.1562% | 23.9902% | 26.0098% | 7.0309% |
| 2009 | -0.0048 | 0.0174 | -0.0097 | 0.0035 | 0.0030 | 9.9665% | 36.4271% | 20.2784% | 7.3303% | 6.2426% | 4.7902% |
| 2010 | -0.0025 | 0.0153 | -0.0108 | 0.0010 | -0.0053 | 5.9796% | 35.9846% | 25.3523% | 2.4451% | 12.5107% | 4.2498% |
| 2011 | -0.0005 | 0.0045 | -0.0123 | 0.0023 | -0.0067 | 1.2628% | 10.8735% | 29.5580% | 5.5003% | 16.0618% | 4.1582% |
| 2012 | -0.0001 | -0.0001 | 0.0026 | -0.0061 | -0.0067 | 0.2420% | 0.3735% | 6.9678% | 16.0107% | 17.6393% | 3.7928% |
| 2013 | -0.0003 | 0.0029 | -0.0033 | -0.0057 | -0.0011 | 1.1712% | 10.1372% | 11.6587% | 19.9788% | 3.9123% | 2.8638% |
| 2014 | 0.0003 | 0.0004 | -0.0024 | -0.0146 | -0.0013 | 0.6947% | 1.1016% | 6.6584% | 39.8050% | 3.5366% | 3.6658% |
| 2015 | 0.0024 | 0.0013 | 0.0011 | -0.0176 | 0.0016 | 6.9664% | 3.6219% | 3.1701% | 50.0000% | 4.5304% | 3.5153% |
| 2016 | 0.0008 | -0.0011 | -0.0004 | -0.0130 | 0.0008 | 2.8614% | 3.8098% | 1.4823% | 44.7080% | 2.7173% | 2.9183% |
| 2017 | -0.0003 | 0.0036 | -0.0004 | -0.0153 | 0.0037 | 0.8498% | 11.2052% | 1.2612% | 47.8890% | 11.4551% | 3.2025% |
| 2018 | -0.0002 | 0.0025 | -0.0017 | -0.0189 | 0.0058 | 0.3559% | 5.8560% | 3.9539% | 44.5655% | 13.7223% | 4.2328% |
| 2019 | -0.0008 | 0.0024 | 0.0003 | -0.0186 | 0.0039 | 1.9160% | 6.0875% | 0.7727% | 46.2400% | 9.8358% | 4.0129% |
| 2020 | -0.0003 | -0.0015 | 0.0067 | -0.0003 | 0.0060 | 1.3062% | 5.9555% | 26.4024% | 1.3251% | 23.5976% | 2.5239% |
| 2021 | -0.0006 | 0.0014 | -0.0046 | -0.0276 | -0.0007 | 0.8487% | 1.9471% | 6.6923% | 39.7324% | 0.9812% | 6.9360% |
| 2022 | -0.0001 | 0.0001 | 0.0067 | -0.0067 | 0.0047 | 0.3702% | 0.3254% | 26.7867% | 27.0621% | 18.7183% | 2.4935% |

**Table A1-2 Structural Variation Analysis of Outpatient Service Provision of Secondary and Tertiary Hospitals**

| **Year** | **VSV** | | **CRSV (%)** | | **DSV（%）** |
| --- | --- | --- | --- | --- | --- |
|  | **Secondary hospitals** | **Tertiary hospitals** | **Secondary hospitals** | **Tertiary hospitals** |  |
| 2008 | 0.0117 | 0.0096 | 16.6985% | 13.6115% | 7.0309% |
| 2009 | -0.0082 | -0.0013 | 17.0278% | 2.7273% | 4.7902% |
| 2010 | -0.0026 | 0.0049 | 6.1574% | 11.5702% | 4.2498% |
| 2011 | -0.0013 | 0.0140 | 3.1174% | 33.6262% | 4.1582% |
| 2012 | -0.0068 | 0.0154 | 15.7345% | 43.0322% | 3.7928% |
| 2013 | -0.0030 | 0.0123 | 13.2791% | 39.8628% | 2.8638% |
| 2014 | 0.0019 | 0.0158 | 5.1093% | 43.0945% | 3.6658% |
| 2015 | 0.0007 | 0.0104 | 2.0648% | 29.6463% | 3.5153% |
| 2016 | 0.0013 | 0.0116 | 4.6057% | 39.8157% | 2.9183% |
| 2017 | 0.0021 | 0.0067 | 6.4370% | 20.9028% | 3.2025% |
| 2018 | -0.0005 | 0.0129 | 1.1246% | 30.4217% | 4.2328% |
| 2019 | -0.0007 | 0.0134 | 1.8440% | 33.3040% | 4.0129% |
| 2020 | -0.0057 | -0.0048 | 22.4135% | 18.9997% | 2.5239% |
| 2021 | -0.0012 | 0.0333 | 1.7455% | 48.0529% | 6.9360% |
| 2022 | -0.0056 | 0.0010 | 22.5678% | 4.1696% | 2.4935% |

**Table A1-3 Structural Variation Analysis of Inpatient Service Provision of Different Types of Primary Healthcare Institutions**

| **Year** | **VSV** | | | | **CRSV (%)** | | | | **DSV (%)** |
| --- | --- | --- | --- | --- | --- | --- | --- | --- | --- |
|  | **First-level hospitals** | **Community health service centers (stations)** | **Township health centers** | **Village clinics** | **First-level hospitals** | **Community health service centers (stations)** | **Township health centers** | **Village clinics** |  |
| 2008 | -0.0003 | 0.0014 | 0.0147 | -0.0004 | 1.0893% | 4.2161% | 45.7839% | 1.2753% | 3.2046% |
| 2009 | -0.0017 | 0.0053 | -0.0006 | 0.0013 | 12.5063% | 39.9189% | 4.4381% | 10.0811% | 1.3245% |
| 2010 | 0.0003 | 0.0018 | -0.0339 | -0.0020 | 0.3601% | 2.4454% | 47.2305% | 2.7695% | 7.1872% |
| 2011 | 0.0027 | 0.0006 | -0.0338 | -0.0019 | 3.7619% | 0.8144% | 47.2778% | 2.7222% | 7.1463% |
| 2012 | 0.0015 | -0.0019 | -0.0075 | -0.0003 | 2.8524% | 3.6033% | 14.4419% | 0.6130% | 5.1677% |
| 2013 | 0.0018 | -0.0006 | -0.0160 | 0.0001 | 3.8589% | 1.4046% | 35.1604% | 0.1457% | 4.5522% |
| 2014 | 0.0012 | -0.0012 | -0.0250 | -0.0002 | 2.1674% | 2.0816% | 44.0202% | 0.3495% | 5.6778% |
| 2015 | 0.0071 | -0.0007 | -0.0113 | -0.0002 | 16.4218% | 1.5274% | 26.1863% | 0.4511% | 4.2989% |
| 2016 | -0.0001 | -0.0009 | -0.0083 | -0.0002 | 0.4505% | 2.9721% | 26.9098% | 0.8008% | 3.0692% |
| 2017 | 0.0023 | 0.0005 | -0.0019 | 0.0000 | 13.5886% | 2.9451% | 11.5407% | 0.2011% | 1.6826% |
| 2018 | -0.0006 | -0.0012 | -0.0109 | -0.0001 | 1.4201% | 2.8023% | 25.0974% | 0.2773% | 4.3539% |
| 2019 | -0.0051 | -0.0010 | -0.0120 | -0.0001 | 9.0728% | 1.6978% | 21.3727% | 0.2199% | 5.6368% |
| 2020 | 0.0054 | -0.0003 | -0.0009 | -0.0003 | 15.8471% | 0.7853% | 2.7489% | 0.8757% | 3.4209% |
| 2021 | -0.0038 | 0.0001 | -0.0188 | 0.0007 | 3.7440% | 0.0909% | 18.7430% | 0.7401% | 10.0510% |
| 2022 | -0.0007 | 0.0006 | 0.0005 | -0.0001 | 1.9420% | 1.5989% | 1.5452% | 0.2601% | 3.4484% |

**Table A1-4 Structural Variation Analysis of Inpatient Service Provision of Secondary and Tertiary Hospitals**

| **Year** | **VSV** | | **CRSV (%)** | | **DSV（%）** |
| --- | --- | --- | --- | --- | --- |
|  | **Secondary hospitals** | **Tertiary hospitals** | **Secondary hospitals** | **Tertiary hospitals** |  |
| 2008 | -0.0066 | -0.0087 | 20.6145% | 27.0209% | 3.2046% |
| 2009 | -0.0034 | -0.0010 | 25.6809% | 7.3747% | 1.3245% |
| 2010 | 0.0138 | 0.0201 | 19.2710% | 27.9235% | 7.1872% |
| 2011 | 0.0043 | 0.0281 | 6.0474% | 39.3763% | 7.1463% |
| 2012 | -0.0162 | 0.0244 | 31.3417% | 47.1476% | 5.1677% |
| 2013 | -0.0061 | 0.0209 | 13.4350% | 45.9954% | 4.5522% |
| 2014 | -0.0020 | 0.0272 | 3.5487% | 47.8326% | 5.6778% |
| 2015 | -0.0094 | 0.0144 | 21.8352% | 33.5782% | 4.2989% |
| 2016 | -0.0058 | 0.0153 | 18.8668% | 50.0000% | 3.0692% |
| 2017 | -0.0064 | 0.0056 | 38.2582% | 33.4663% | 1.6826% |
| 2018 | -0.0089 | 0.0218 | 20.4029% | 50.0000% | 4.3539% |
| 2019 | -0.0099 | 0.0282 | 17.6368% | 50.0000% | 5.6368% |
| 2020 | -0.0156 | 0.0117 | 45.5901% | 34.1529% | 3.4209% |
| 2021 | -0.0276 | 0.0494 | 27.5130% | 49.1690% | 10.0510% |
| 2022 | -0.0165 | 0.0162 | 47.7979% | 46.8558% | 3.4484% |
